# Supplementary material for: Deciphering Rhizosphere Microbiome Assembly of Castanea henryi in Plantation and Natural Forest
Source: Microorganisms. 2021 Dec 26;10(1):42. doi: 10.3390/microorganisms10010042 (PMC8779262; doi:10.3390/microorganisms10010042)
Supplement: Supplementary file 1 [file microorganisms-10-00042-s001.zip › Table S1.pdf]

Table S1. Topological properties of the empirical molecular ecological networks (MENs) of microbial communities.

	TRY	TRB	TRC	TTX
**Total nodes**	817	815	832	818
**Total links**	1967	1748	2021	2165
**Average degree (avgK)**	4.815	4.29	4.858	5.293
**Average path distance (GD)**	10.303	14.019	13.073	9.974
**Average clustering coefficient (avgCC)**	0.437	0.362	0.429	0.419
**Positive correlation**	1132	1126	1335	1572
**Negative correlation**	835	622	686	593
**Rare taxa**	75.15%	74.36%	67.79%	67.48%
**Moderate taxa**	22.03%	21.10%	26.68%	26.77%
**Abundance taxa**	2.82%	4.54%	5.53%	5.75%

TRY, TRC, and TRB, three cultivated varieties of plantation; TTX, natural forest.
