# Supplementary figures and images for: Deciphering Rhizosphere Microbiome Assembly of Castanea henryi in Plantation and Natural Forest
Source: Microorganisms. 2021 Dec 26;10(1):42. doi: 10.3390/microorganisms10010042 (PMC8779262; doi:10.3390/microorganisms10010042)

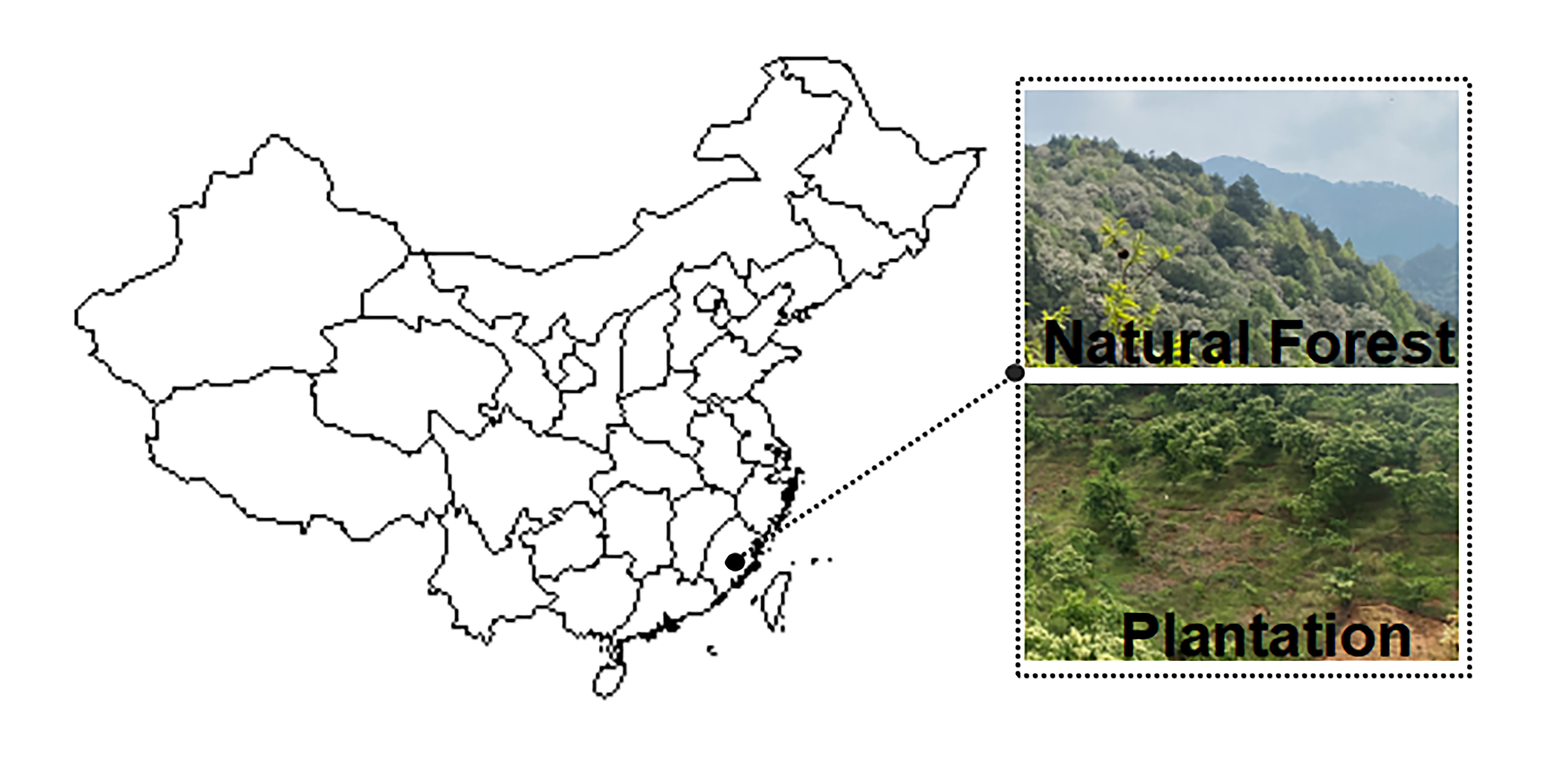

Supplement: Supplementary file 1 [file microorganisms-10-00042-s001.zip › Figure S1.tif]

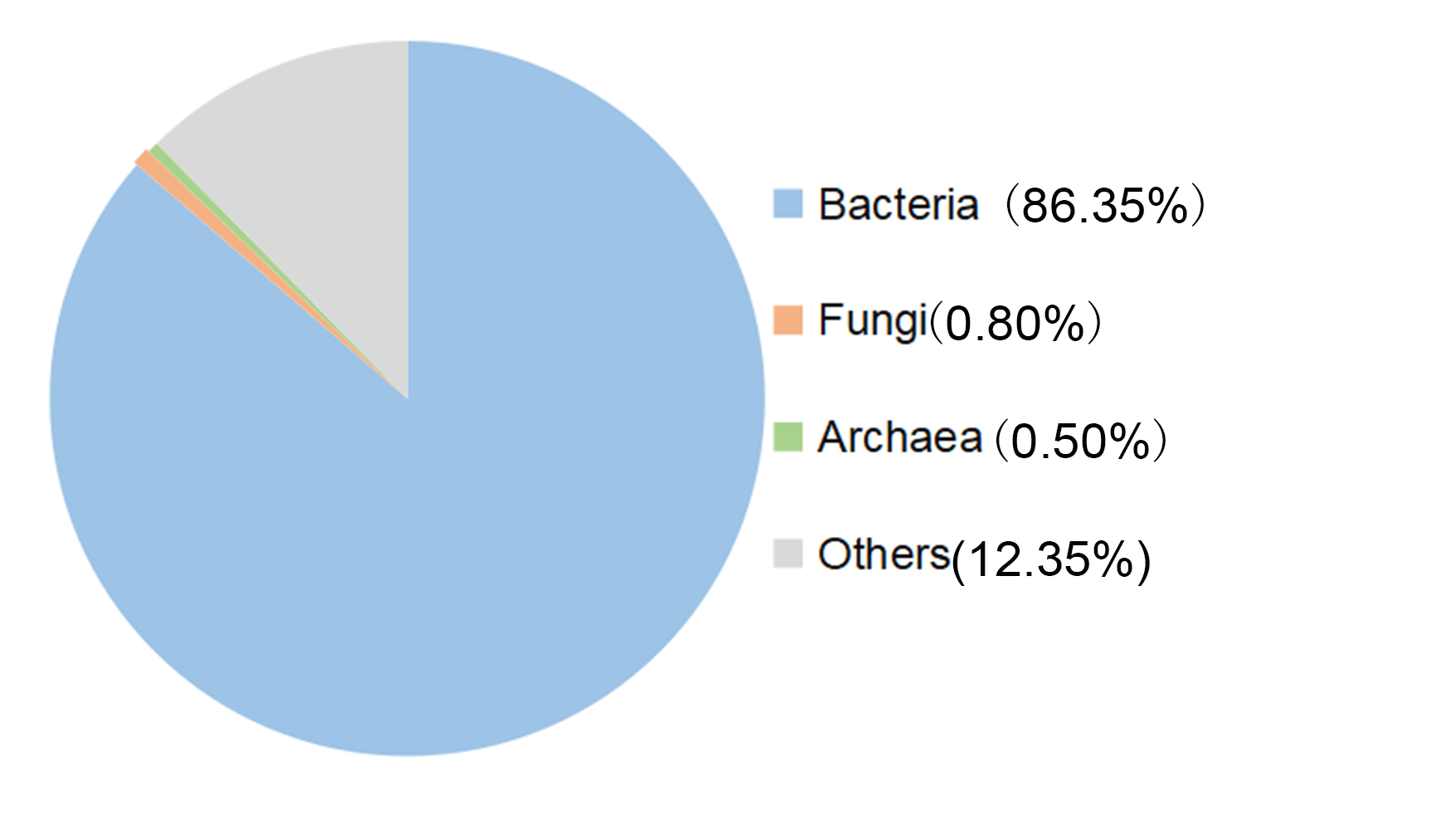

Supplement: Supplementary file 1 [file microorganisms-10-00042-s001.zip › Figure S2 .tif]

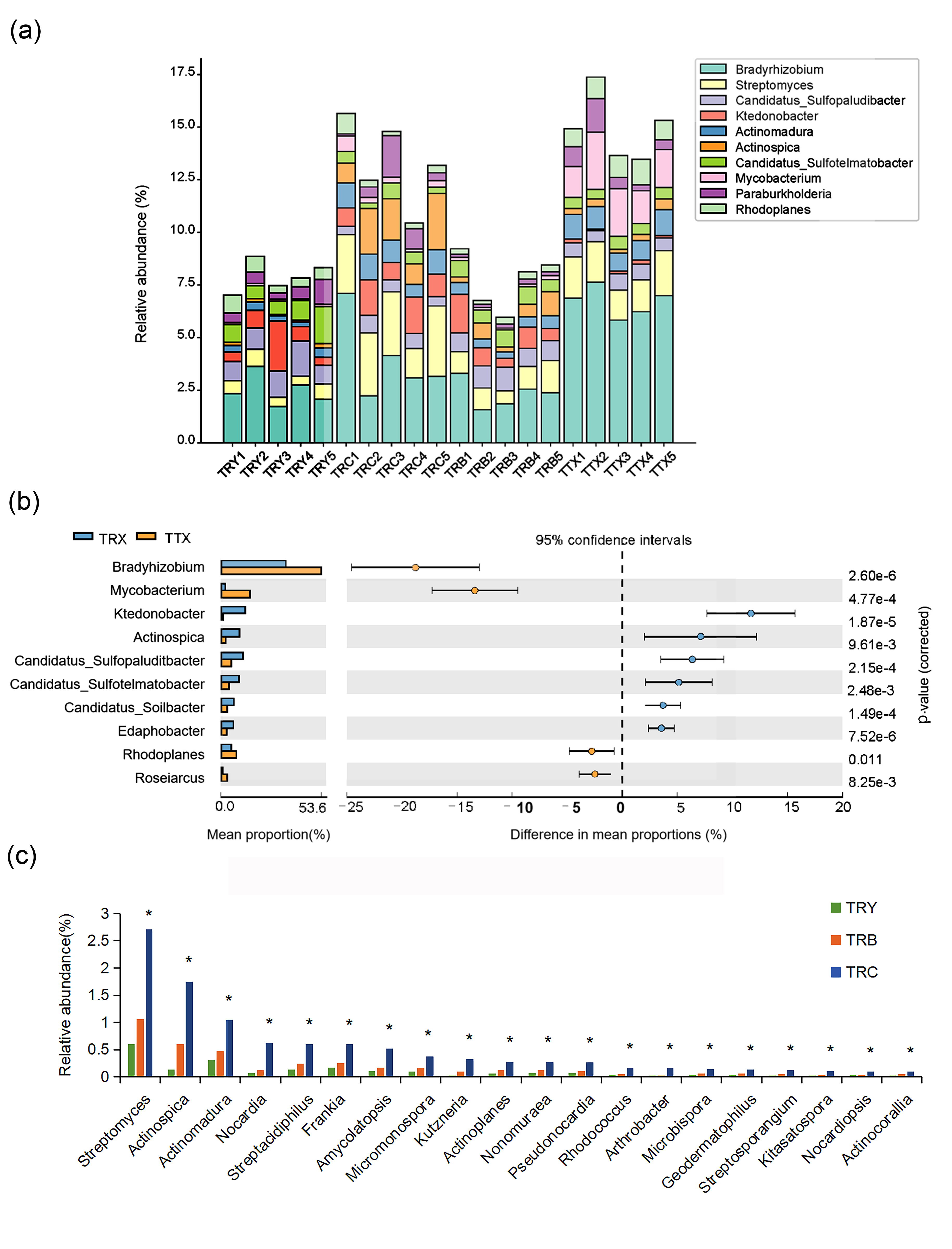

Supplement: Supplementary file 1 [file microorganisms-10-00042-s001.zip › Figure S3.tif]

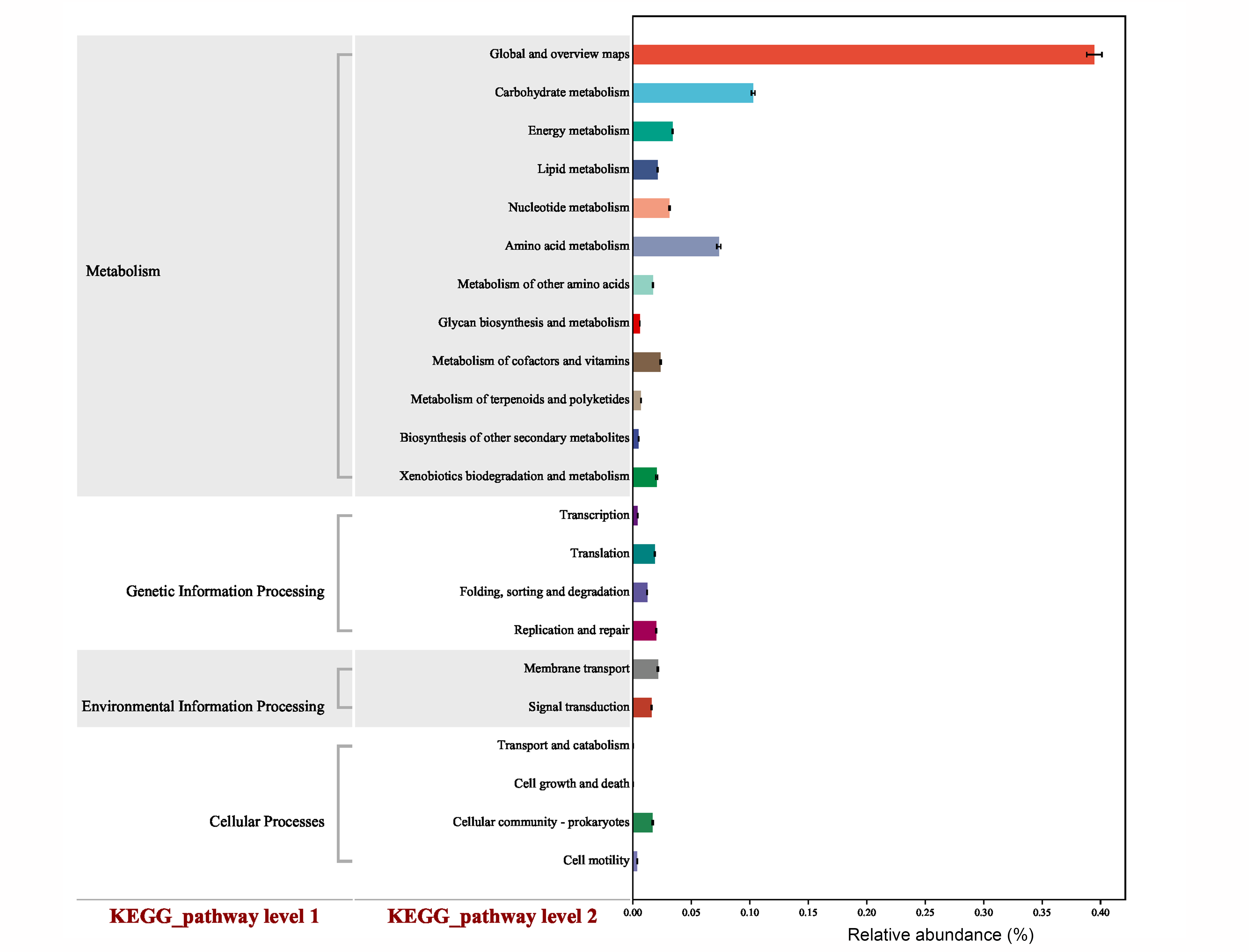

Supplement: Supplementary file 1 [file microorganisms-10-00042-s001.zip › Figure S4.tif]

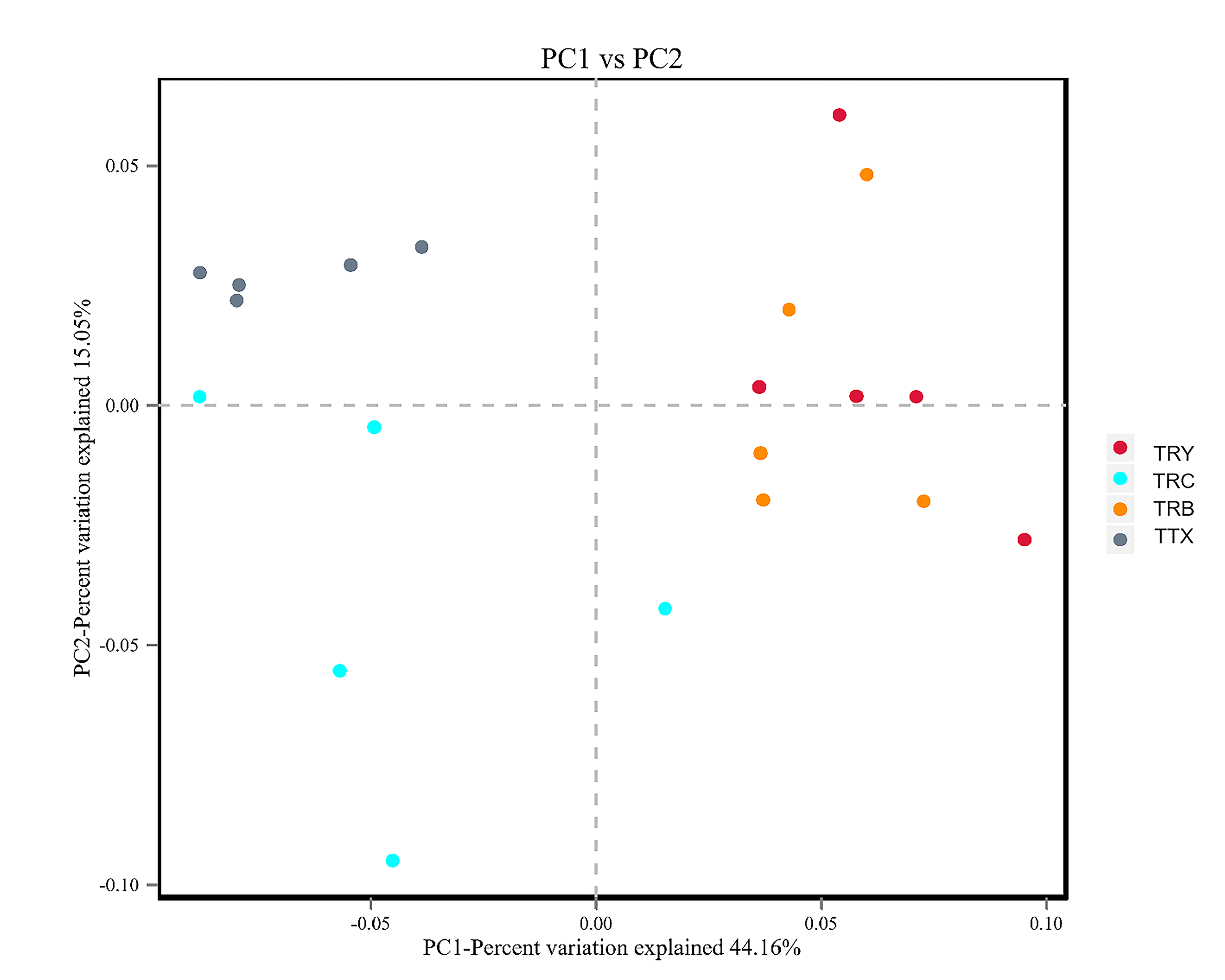

Supplement: Supplementary file 1 [file microorganisms-10-00042-s001.zip › Figure S5.tif]

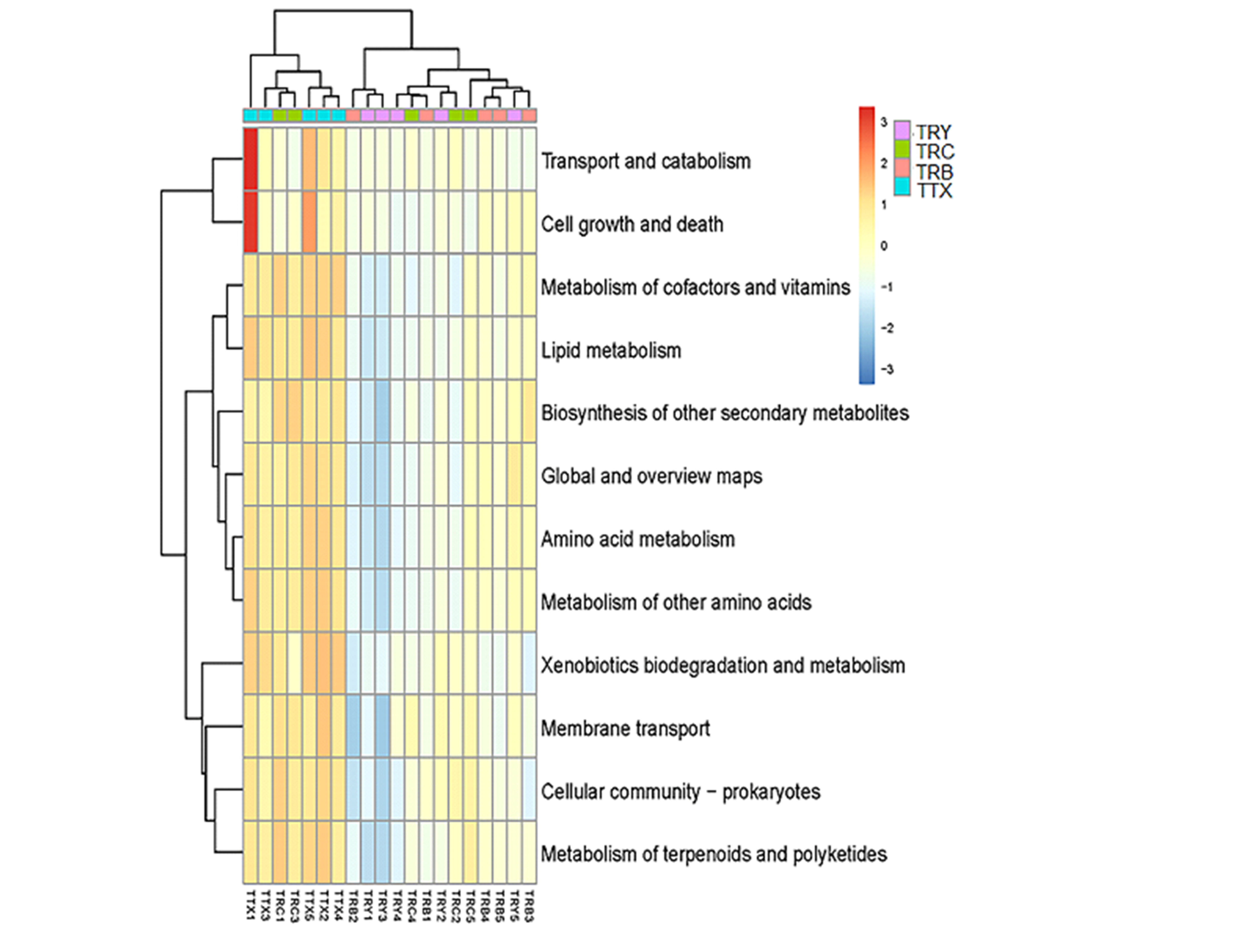

Supplement: Supplementary file 1 [file microorganisms-10-00042-s001.zip › Figure S6 .tif]

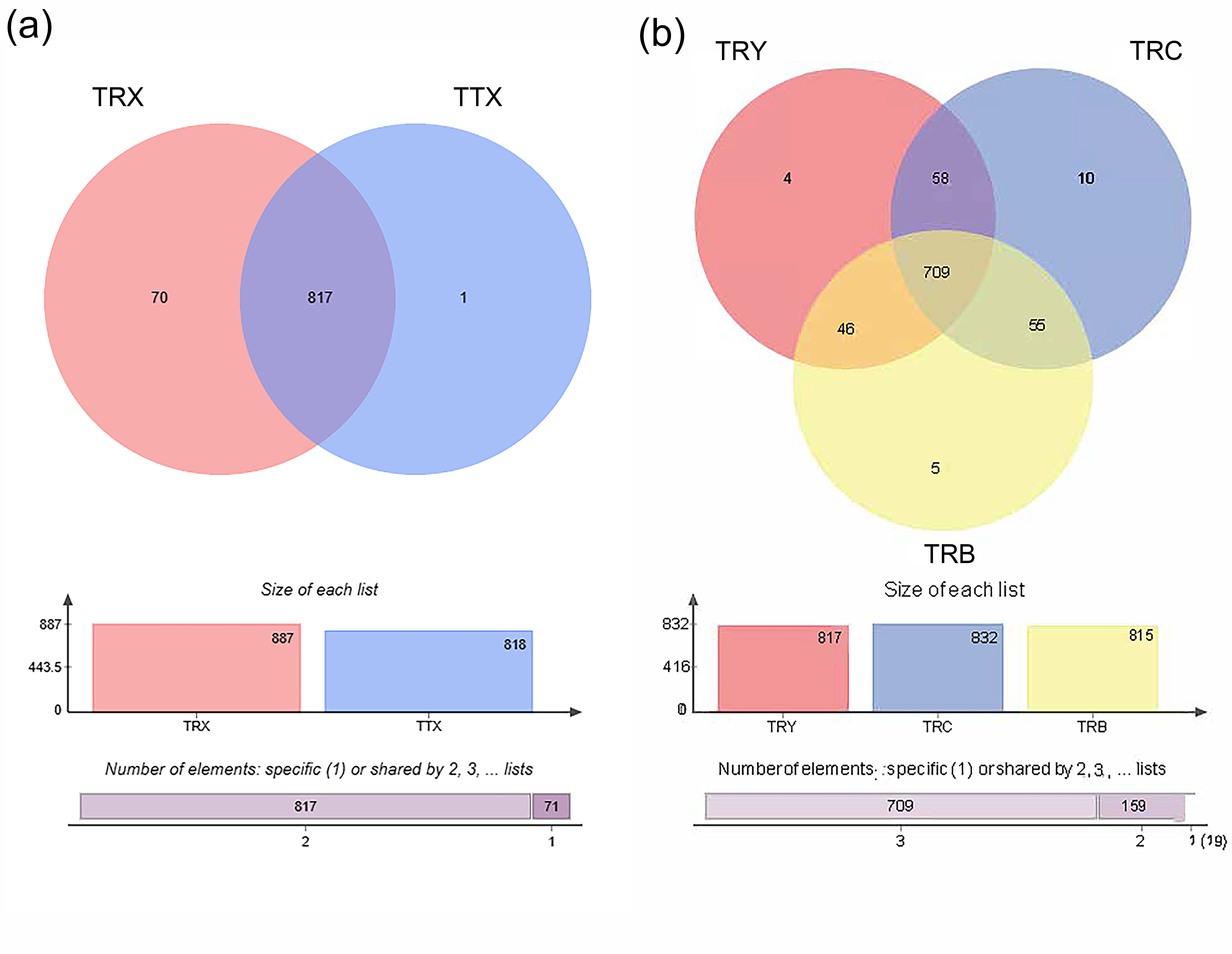

Supplement: Supplementary file 1 [file microorganisms-10-00042-s001.zip › Figure S7.tif]
